# A spectral Fletcher-Reeves conjugate gradient method with integrated strategy for unconstrained optimization and portfolio selection

**DOI:** 10.1371/journal.pone.0320416

**Published:** 2025-04-25

**Authors:** Nasiru Salihu, Sulaiman M. Ibrahim, P. Kaelo, Issam A.R. Moghrabi, Elissa Nadia Madi

**Affiliations:** 1 Department of Mathematics, Faculty of Sciences, Modibbo Adama University, Yola, Nigeria; 2 School of Quantitative Sciences, Universiti Utara Malaysia, Sintok, Kedah, Malaysia; 3 Faculty of Education and Arts, Sohar University, Sohar, Oman; 4 Department of Mathematics, University of Botswana, Private Bag UB00704, Gaborone, Botswana; 5 Information Systems and Technology Department, Kuwait Technical College, Abu-Halifa, Kuwait; 6 Faculty of Informatics and Computing, Universiti Sultan Zainal Abidin, Terengganu, Malaysia; Khalifa University of Science and Technology, UNITED ARAB EMIRATES

## Abstract

The spectral conjugate gradient (SCG) technique is highly efficient in addressing large-scale unconstrained optimization challenges. This paper presents a structured SCG approach that combines the Quasi-Newton direction and an extended conjugacy condition. Drawing inspiration from the Fletcher-Reeves conjugate gradient (CG) parameter, this method is tailored to improve the general structure of the CG approach. We rigorously establish the global convergence of the algorithm for general functions, using criteria from a Wolfe-line search. Numerical experiments performed on some unconstrained optimization problems highlight the superiority of this new algorithm over certain CG methods with similar characteristics. In the context of portfolio selection, the proposed method extended to address the problem of stock allocation, ensuring optimized returns while minimizing risks. Empirical evaluations demonstrate the efficiency of the method, demonstrating significant improvements in computational efficiency and optimization outcomes.

## 1 Introduction

In this study, we explore the theoretical analysis and numerical performance of nonlinear conjugate gradient (CG) algorithm for solving minimization problem of the form:


$$\begin{array}{ccc} \min f(x),\;x\in {\mathbb{R}}^{n}, & & \end{array}$$ (1.1)

where $f:{\mathbb{R}}^{n}\to \mathbb{R}$ is a smooth function with *g*(*x*) = ∇*f*(*x*) as its gradient [1]. The CG algorithm is one of the most widely used line search procedures for solving (1.1) due to its favorable theoretical properties and robust computational performance on large-scale minimization functions [2–4]. Recently, numerous studies have extended CG methods to solve real-world application problems, such as portfolio selection in the context of portfolio optimization. Portfolio optimization is a critical area of research in finance, aiming to balance risk and return while adhering to various constraints. In this domain, the mathematical formulation of portfolio optimization often leads to large-scale unconstrained optimization problems, which require efficient numerical methods [5,6].

Usually line search is the most important component in the CG iterative scheme, beginning with a starting point $x_{0}\in {\mathbb{R}}^{n}$, the algorithm computes a sequence of iterative points $\left\{x_{k}\right\}$ via


$$\begin{array}{ccc} \begin{array}{c} x_{k}=x_{k-1}+\alpha_{k}d_{k-1},\;k\geq 0, \end{array} & & \end{array}$$ (1.2)

with the step-size $\alpha_{k}$ computed via a suitable line search procedure such as the standard Wolfe strategy that requires $\alpha_{k}$ to satisfy


$$\begin{array}{ccc} f(x_{k}+\alpha_{k}d_{k}) & \leq f(x_{k})+\delta \alpha_{k}g_{k}^{T}d_{k}, & \end{array}$$ (1.3)

and


$$\begin{array}{ccc} g{\left(x_{k}+\alpha_{k}d_{k}\right)}^{T}d_{k} & \geq \sigma g_{k}^{T}d_{k}. & \end{array}$$ (1.4)

The inequality (1.4) contains the curvature condition that assures a sufficient increase of *g*(*x*). However, the $\alpha_{k}$ might not converge to the minimum of the cost functions, potentially affecting the overall convergence findings. To avoid this instances, a modified curvature condition was therefore defined as


$$\begin{array}{ccc} |g{\left(x_{k}+\alpha_{k}d_{k}\right)}^{T}d_{k}|\leq -\sigma g_{k}^{T}d_{k}, & & \end{array}$$ (1.5)

and when combined with (1.3) produces what is known as the strong Wolfe (SW) line search, with *δ* < *σ* < 1, where $0<\delta <\frac{1}{2}.$

The second key element of the CG algorithm is the search direction $d_{k}$ and is computed using


$$\begin{array}{ccc} d_{0}=-g_{0},\;d_{k+1}=-g_{k+1}+\beta_{k}d_{k},\;\forall k\geq 0, & & \end{array}$$ (1.6)

with the scalar parameter $\beta_{k}$ being the CG coefficient that distinguishes between different CG formulas [7]. Some of the famous and classical CG schemes include Fletcher and Reeves (FR)[8], Polak, Ribière and Polyak (PRP) [9,10], Hestenes and Stiefel (HS) [11], and Dai and Yuan (DY) [12], with the following formulas for $\beta_{k}$:


$$\begin{array}{ccc} \beta_{k}^{FR}=\frac{∥g_{k+1}{∥}^{2}}{∥g_{k}{∥}^{2}},\;\beta_{k}^{PRP}=\frac{g_{k+1}^{T}y_{k}}{∥g_{k}{∥}^{2}},\;\beta_{k}^{HS}=\frac{g_{k+1}^{T}y_{k}}{d_{k}^{T}y_{k}},\;\beta_{k}^{DY}=\frac{∥g_{k+1}{∥}^{2}}{d_{k}^{T}y_{k}}, & & \end{array}$$ (1.7)

where $y_{k}=g_{k+1}-g_{k}$ and  ∥⋅∥ represents the $\ell_{2}$ norm [13]. It is generally believed that the classical PRP and HS CG algorithms are very efficient in practical computations. This is due to the restart feature associated with these methods when jamming occurs. However, these methods are not guaranteed to converge for general functions, with the PRP formula lacking to produce a descent direction [14].

Thus, the standard conjugacy condition $y_{k}^{T}d_{k+1}=0$ is often used in the convergence analysis of CG algorithms. The CG methods that make use of this technique depend largely on exactly computing $\alpha_{k}$, and this requirement is costly for large-scale models [15]. Therefore, Perry [16] incorporated a second-order information into $y_{k}^{T}d_{k+1}=0$ to implement many CG schemes that require (1.3) and (1.5) in their convergence. This is made possible by the fact that if the present iterate $x_{k+1}$ is in the vicinity of the local minimizer, and the cost function behaves closely like a quadratic function, then the quasi-Newton direction is the suitable search direction to follow [17]. Later, Dai and Liao [18] considered the following general conjugacy condition


$$\begin{array}{ccc} d_{k+1}^{T}y_{k}=-tg_{k+1}^{T}s_{k}, & & \end{array}$$ (1.8)

from which they obtained a new formula for the CG method as


$$\begin{array}{ccc} \beta_{k}^{DL}=\beta_{k}^{HS}-t\frac{g_{k+1}^{T}s_{k}}{d_{k}^{T}y_{k}}, & & \end{array}$$ (1.9)

where $s_{k}=x_{k+1}-x_{k}$. To prove the convergence results for general functions, they specified (1.9) as


$$\begin{array}{ccc} \beta_{k}^{DL*}=\max\{\beta_{k}^{HS},0\}-t\frac{g_{k+1}^{T}s_{k}}{d_{k}^{T}y_{k}},\;t>0. & & \end{array}$$ (1.10)

By considering the technique in [18], the authors of [19] introduced an extension of the PRP method in the form of


$$\begin{array}{ccc} \beta_{k}^{EPRP}=\beta_{k}^{PRP}-t\frac{g_{k+1}^{T}d_{k}}{{∥g_{k}∥}^{2}},\;t>0. & & \end{array}$$ (1.11)

It is obvious that when *t* = 0, (1.9) and (1.11) will reduce to the classical HS and PRP CG methods defined in (1.7). Similarly, taking the value of *t* as $2{∥y_{k}∥}^{2}∕(d_{k}^{T}y_{k})$ and ${∥g_{k+1}∥}^{2}∕(d_{k}^{T}y_{k}),$ Hager and Zhang [20] and Andrei [21] extended the above idea to construct accelerated versions of [18] as


$$\begin{array}{ccc} \beta_{k}^{N}=\beta_{k}^{HS}-2\frac{{∥y_{k}∥}^{2}}{{\left(d_{k}^{T}y_{k}\right)}^{2}}g_{k+1}^{T}d_{k}, & & \end{array}$$ (1.12)

and


$$\begin{array}{ccc} \beta_{k}^{A}=\beta_{k}^{DY}-\frac{{∥g_{k+1}∥}^{2}}{{\left(d_{k}^{T}y_{k}\right)}^{2}}g_{k+1}^{T}s_{k}, & & \end{array}$$ (1.13)

respectively. The formula (1.12) satisfies the sufficient descent condition, and is globally convergent for general functions under the restriction


$$\begin{array}{ccc} \beta_{k}^{N+}=\max\left\{\beta_{k}^{N},\frac{-1}{∥d_{k}∥\min\{\eta ,∥g_{k}∥\}}\right\}. & & \end{array}$$ (1.14)

Experimental results has demonstrated the efficiency and robustness of the method.

Spectral CG directions are other important modifications of the CG directions which aim to improve the theoretical features of classical CG formula as well as their numerical performance. The techniques are based on studies presented by Barzilai and Borwein[22], and Raydan [23]. The method has the structure


$$d_{k+1}=-\theta_{k}g_{k+1}+\beta_{k}d_{k},\;\forall k\geq 0,$$

where $\theta_{k}$ denote the spectral parameter. For nice contribution on the subject, the reader is referred to [24–29]. Applying this technique, Liu et al. [30], using $\beta_{k}^{FR}$, constructed the spectral parameter


$$\theta_{k}=-\frac{g_{k}^{T}d_{k}}{g_{k}^{T}g_{k}}+\beta_{k}\frac{g_{k+1}^{T}d_{k}}{g_{k+1}^{T}g_{k+1}},$$

and examined the convergence by assuming that $\beta_{k}$ is bounded, further illustrating the computational efficiency with a set of minimization functions. However, these spectral parameters do not consider conjugacy condition given by (1.8), and Quasi-Newton technique in its formulation, a feature that makes CG method possess a quadratic convergence property. Following the approach suggested in [21], Faramarzi and Amini [31] constructed a double truncated structured spectral parameter using some features of the famous quasi-Newton procedure and the standard secant equation by Jian et al. [32]. To guarantee that $d_{k}$ defined in the algorithm possesses the sufficient descent property, specifically


$$\begin{array}{ccc} d_{k+1}^{T}g_{k+1}\leq -\tau {∥g_{k+1}∥}^{2},\;\forall k\geq 0, & & \end{array}$$ (1.15)

they used the truncated double bounded property, that is, $\theta_{K}$ is bounded below and above. Using the above technique with modified secant equation, [33] also suggested a similar spectral structure. These modifications show that, the spectral methods in [31–33] all possess (1.15) and are globally convergent under the lower bound and upper bound respectively.

Inspired by the above discussion and considering the extended conjugacy condition with the excellent theoretical features of quasi-Newton schemes, this study suggests a spectral FR CG algorithm for solving (1.1), particularly, when the problems are of large dimensions. The proposed search direction $d_{k}$ satisfies the descent condition and converges globally via strong Wolfe conditions. Using a set of benchmark functions, our computational experiments demonstrate that the method is very promising compared to some modified CG algorithms. The subsequent section details every formulation step of our proposed spectral formula with its algorithm. For Sect 3, the convergence results is established under suitable assumptions, and we demonstrate the numerical performance of the proposed method on benchmark functions with application to portfolio selection in Sect 4. The final section presents our conclusions.

## 2 Spectral algorithm and motivation

To provide a better CG algorithm, the spectral parameter $\theta_{k}$ can be incorporated into the structure of the direction of search $d_{k}$ in (1.6) such that it satisfies (1.8). To achieve this, consider the directions


$$\begin{array}{ccc} d_{k+1}=-\theta_{k+1}g_{k+1}+\beta_{k}^{FR}d_{k},\;\forall k\geq 0, & & \end{array}$$ (2.1)

and


$$\begin{array}{ccc} d_{k+1}=-B_{k+1}^{-1}g_{k+1}, & & \end{array}$$ (2.2)

with $B_{k+1}$ being the approximation of the Hessian matrix $\nabla^{2}f(x_{k+1}).$ The property that ensures quadratic convergence of a CG method, assuming that $B_{k+1}$ exists, is characterized by the secant equation


$$\begin{array}{ccc} B_{k+1}s_{k}=y_{k}. & & \end{array}$$ (2.3)

Equating (2.1) with (2.2) gives


$$-B_{k+1}^{-1}g_{k+1}=-\theta_{k+1}g_{k+1}+\beta_{k}^{FR}d_{k}.$$

Multiplying the above equation by $s_{k}^{T}B_{k+1}$, and using (2.3), we have


$$\begin{array}{llll} -s_{k}^{T}g_{k+1} & =-\theta_{k+1}s_{k}^{T}B_{k+1}g_{k+1}+\beta_{k}^{FR}s_{k}^{T}B_{k+1}d_{k}\; & & \; \end{array}$$

which gives that


$$\begin{array}{llll} -s_{k}^{T}g_{k+1} & =-\theta_{k+1}y_{k}^{T}g_{k+1}+\beta_{k}^{FR}y_{k}^{T}d_{k}.\; & & \; \end{array}$$

Simplifying in terms of $\theta_{k+1}$ gives


$$\begin{array}{ccc} \theta_{k+1}^{SQM}=\frac{s_{k}^{T}g_{k+1}}{y_{k}^{T}g_{k+1}}+\beta_{k}^{FR}\frac{y_{k}^{T}d_{k}}{y_{k}^{T}g_{k+1}}. & & \end{array}$$ (2.4)

Similarly, multiplying (2.1) with $y_{k}^{T}$, yields


$$y_{k}^{T}d_{k+1}=-\theta_{k+1}y_{k}^{T}g_{k+1}+\beta_{k}^{FR}y_{k}^{T}d_{k}.$$

Equating with (1.8) gives


$$-ts_{k}^{T}g_{k+1}=-\theta_{k+1}y_{k}^{T}g_{k+1}+\beta_{k}^{FR}y_{k}^{T}d_{k}.$$

Re- arranging, implies


$$\begin{array}{ccc} \theta_{k+1}^{SDL}=\frac{ts_{k}^{T}g_{k+1}}{y_{k}^{T}g_{k+1}}+\beta_{k}^{FR}\frac{y_{k}^{T}d_{k}}{y_{k}^{T}g_{k+1}}. & & \end{array}$$ (2.5)

**Remark 2.1.** *It follows from (2.5) that if* *t* = 1, *then $\theta_{k+1}^{SDL}=\theta_{k+1}^{SQM}$, which implies that, $d_{k+1}$ inherited the excellent convergence condition of the quasi-Newton algorithm and further satisfy the generalized D-L conjugacy property.*

Therefore, to ensure the sufficient descent condition, the optimal spectral (DQSFR) can be determined as


$$\begin{array}{ccc} \theta_{k+1}^{DQSFR}=\max\left\{1,t\frac{s_{k}^{T}g_{k+1}}{y_{k}^{T}g_{k+1}}+\beta_{k}^{FR}\frac{y_{k}^{T}d_{k}}{y_{k}^{T}g_{k+1}}\right\}. & & \end{array}$$ (2.6)

The DQSFR algorithm is given below.

**Algorithm 1.**

**Step 1:** Given $x_{0}\in {\mathbb{R}}^{n}$ and 0 < *δ* < *σ* < 1, set $d_{0}=-g_{0},$ *k* = 0 and $\alpha_{0}=1$.

**Step 2:** Check: If $∥g_{k}∥\leq 𝜖$, then terminate. Else

**Step 3:** Select $\alpha_{k}>0$ along the direction $d_{k}$ such that (1.3) and (1.5) are satisfied.

**Step 4:** Let $x_{k+1}=x_{k}+\alpha_{k}d_{k};$ Compute $\theta_{k+1}^{DQSFR}$ by (2.6) and $\beta_{k}^{FR}$ from (1.7). Set *k* = *k* + 1.

**Step 5:** Compute $d_{k}$ from (2.1).

**Step 6:** Go to Step 2 with the next *k*.

Next we show that the spectral method satisfies the sufficient descent condition.

**Theorem 2.2.** *Suppose $\theta_{k+1}$ follows from* (2.6)*, with DQSFR Algorithm generating sequences $\left\{g_{k}\right\}$ and $\left\{d_{k}\right\}.$ Then there exists a constant* *ρ* > 0 *satisfying*


$$\begin{array}{ccc} d_{k+1}^{T}g_{k+1}\leq -\rho {∥g_{k+1}∥}^{2},\;\forall k\geq 0. & & \end{array}$$ (2.7)

*Proof.* Pre-multiplying (2.1) by $g_{k+1}^{T}$, we obtain


$$\begin{array}{ccc} g_{k+1}^{T}d_{k+1}=-\theta_{k+1}{∥g_{k+1}∥}^{2}+\beta_{k}^{FR}g_{k+1}^{T}d_{k}. & & \end{array}$$ (2.8)

Obtaining $\beta_{k}^{FR}$ from (1.7) and using it with (2.8), we get


$$\begin{array}{cccc} g_{k+1}^{T}d_{k+1} & =-\theta_{k+1}{∥g_{k+1}∥}^{2}+\frac{∥g_{k+1}{∥}^{2}}{∥g_{k}{∥}^{2}}g_{k+1}^{T}d_{k}. & & \end{array}$$

From the definition of $\theta_{k+1}$ in (2.6), we know that $\theta_{k+1}\geq 1.$ Therefore, we have


$$\begin{array}{ccc} g_{k+1}^{T}d_{k+1}\leq -{∥g_{k+1}∥}^{2}+\frac{∥g_{k+1}{∥}^{2}}{∥g_{k}{∥}^{2}}g_{k+1}^{T}d_{k}. & & \end{array}$$ (2.9)

Al-Baali [34] showed that (2.9) satisfies (2.7) using the induction hypothesis obtained from [35, Theorem 4.2]


$$\begin{array}{ccc} -\frac{1}{1-\sigma }\leq \frac{g_{k}^{T}d_{k}}{{∥g_{k}∥}^{2}}\leq \frac{2\sigma -1}{1-\sigma }, & & \end{array}$$ (2.10)

where $\sigma <\frac{1}{2}.$ Using (2.9) implies


$$\begin{array}{cccc} \frac{g_{k+1}^{T}d_{k+1}}{{∥g_{k+1}∥}^{2}} & \leq -1+\frac{g_{k+1}^{T}d_{k}}{{∥g_{k}∥}^{2}}. & & \end{array}$$

Applying (1.5), we have


$$-1+\sigma \frac{g_{k}^{T}d_{k}}{{∥g_{k}∥}^{2}}\leq \frac{g_{k+1}^{T}d_{k+1}}{{∥g_{k+1}∥}^{2}}\leq -1-\sigma \frac{g_{k}^{T}d_{k}}{{∥g_{k}∥}^{2}}.$$

Consequently, using (2.10) on the last term of the above inequality yields


$$-1-\frac{\sigma }{1-\sigma }\leq \frac{g_{k+1}^{T}d_{k+1}^{FR}}{{∥g_{k+1}∥}^{2}}\leq -1+\frac{\sigma }{1-\sigma }.$$

Hence, denoting $\rho =(1-\frac{\sigma }{1-\sigma }),$ we get


$$g_{k+1}^{T}d_{k+1}\leq -\rho {∥g_{k+1}∥}^{2}.$$

∎

Since $\theta_{k+1}\geq 1,$ this implies that the descent condition (2.7) holds when $\rho =\theta_{k+1}-\frac{\sigma }{1-\sigma }$ with *σ* ∈ (0, 0.4]. This motivated the derivation of DQSFR formula in (2.6).

## 3 Convergence analysis

The convergence proof of the SCG method relies on certain assumptions, such as the Lipschitz continuity of the gradient of the target function. While these assumptions are standard in optimization theory, they may not always hold in practical applications, particularly for non-smooth functions encountered in real-world problems. This limitation can affect the theoretical guarantees of convergence and may restrict the method’s direct applicability in such cases.

In this study, we consider the following assumptions that are crucial in achieving the convergence results of the proposed method.

**Assumption 3.1.**

*1. Let f(x) represent a function and $S_{0}=\{x\in {\mathbb{R}}^{n}:f(x_{0})\geq f(x)\}$ define a level set where $x_{0}\in {\mathbb{R}}^{n}$. Then, f(x) is bounded below on $S_{0}$ and there exists a positive constant b satisfying*
$$∥x∥\leq b,\;\forall x\in S_{0}.$$
*2.* *The function *f* is a smooth function in some neighbourhood Γ of $S_{0}$, and its gradient is Lipschitz continuous. This implies, for some *L*, we have*
  ∥g(x)−g(y)∥≤L∥x−y∥, ∀ ⁡x,y∈Γ,
*where *L* denotes a positive constant.*
*Based on Assumption (3.1), there is a positive constant *γ* satisfying:*
$$\begin{array}{llll} ∥g(x)∥ & \leq \gamma ,\;\forall x\in S_{0}.\; & & \; \end{array}$$

Using sufficient descent criteria (2.7) and the line search technique (1.3), it is clear to see that $\left\{f(x_{k})\right\}$ decreases monotonically. Owing to the fact that the function *f* is bounded from below, there is a parameter f^*^, satisfying


$$\begin{array}{ccc} \underset{k\to \infty }{\lim}f(x_{k})=f^{*}, & & \end{array}$$ (3.1)

where f^*^ is a constant. Thus, Lemma 3.2 that follows by [36] is very vital in the convergence analysis of the new method.

**Lemma 3.2.** *Let the sequence $\left\{x_{k}\right\}$ be generated by the proposed DQSFR Algorithm where $d_{k}$ is a descent spectral direction with a chosen $\alpha_{k}$ that fulfills the weak Wolfe conditions. Then*


$$\overset{\infty }{\underset{k=1}{\sum }}\frac{{\left(g_{k}^{T}d_{k}\right)}^{2}}{{∥d_{k}∥}^{2}}<\infty .$$

Note that $\alpha_{k}$ that satisfies (1.5) also satisfies (1.4). Hence, using strong Wolfe search condition, we can see that Lemma 3.2 also holds true.

*Proof.* From Assumption 3.1 and curvature condition (1.4), we get


$$(\sigma -1)d_{k}^{T}g_{k}\leq {\left(g_{k+1}-g_{k}\right)}^{T}d_{k}\leq ∥g_{k+1}-g_{k}∥∥d_{k}∥\leq L\alpha_{k}∥d_{k}{∥}^{2}.$$

Then, we have


$$\alpha_{k}\geq \frac{\left(\sigma -1\right)}{L}\frac{\left(d_{k}^{T}g_{k}\right)}{∥d_{k}{∥}^{2}}.$$

Using relation (1.3), we have


$$f(x_{k})-f(x_{k}+\alpha_{k}d_{k})\geq \delta \frac{\left(1-\sigma \right)}{L}\frac{{\left(d_{k}^{T}g_{k}\right)}^{2}}{∥d_{k}{∥}^{2}}.$$

From the above inequality, and Assumption 3.1, we have


$$\overset{\infty }{\underset{k=1}{\sum }}\frac{{\left(g_{k}^{T}d_{k}\right)}^{2}}{{∥d_{k}∥}^{2}}<\infty .$$

∎

**Lemma 3.3.** *Let the sequences $\left\{x_{k}\right\}$ and $\left\{d_{k}\right\}$ be generated via Algorithm 1, where $\theta_{k+1}>\sigma$, and Assumption 3.1 holds true. Suppose the DQSFR direction $d_{k}$ satisfies the descent condition and the step size is chosen to fulfill the SW conditions (1.3) and (1.5). Then, either*


$$\begin{array}{ccc} \underset{k\to \infty }{\mathrm{lim inf}}∥g_{k}∥=0 & & \end{array}$$ (3.2)

*or*


$$\begin{array}{ccc} \overset{\infty }{\underset{k=1}{\sum }}\frac{{∥g_{k}∥}^{4}}{{∥d_{k}∥}^{2}}<\infty & & \end{array}$$ (3.3)

*holds.*

*Proof.* Since $\theta_{k+1}>\sigma$ and *σ* ∈ (0, 0.4], then, $d_{k}$ generated by Algorithm 1 is a descent direction for all *k*, i.e., $g_{k+1}^{T}d_{k+1}<0.$ Applying (2.1), we get


$$\begin{array}{cccc} {\left(\beta_{k}^{FR}\right)}^{2}{∥d_{k}∥}^{2} & ={∥d_{k+1}+\theta_{k+1}g_{k+1}∥}^{2} & & \\ & ={∥d_{k+1}∥}^{2}+2\theta_{k+1}g_{k+1}^{T}d_{k+1}+\theta_{k+1}^{2}{∥g_{k+1}∥}^{2} & & \\ & \leq {∥d_{k+1}∥}^{2}+\theta_{k+1}^{2}{∥g_{k+1}∥}^{2}. & & \end{array}$$

So, we obtain


$$\begin{array}{ccc} {∥d_{k+1}∥}^{2}\geq {\left(\beta_{k}^{FR}\right)}^{2}{∥d_{k}∥}^{2}-\theta_{k+1}^{2}{∥g_{k+1}∥}^{2}. & & \end{array}$$ (3.4)

Also, since the direction $d_{k+1}$ is descent, by (2.1), we further obtain that


$$g_{k+1}^{T}d_{k+1}-\beta_{k}^{FR}g_{k+1}^{T}d_{k}=-\theta_{k+1}{∥g_{k+1}∥}^{2}.$$

This implies,


$$\begin{array}{ccc} |\beta_{k}^{FR}||g_{k+1}^{T}d_{k}|+|g_{k+1}^{T}d_{k+1}|\geq \theta_{k+1}{∥g_{k+1}∥}^{2}. & & \end{array}$$ (3.5)

Furthermore, taking into account the fact that the highest value *σ* would be able to achieve in (1.5) when equality holds in (2.9) with $\theta_{k+1}=1$, we get that $\sigma_{max}=0.4.$ Therefore, (1.5) can be written as


$$|g_{k+1}^{T}d_{k}|\leq -0.4g_{k}^{T}d_{k}=0.4|g_{k}^{T}d_{k}|.$$

Combining with (3.5) gives


$$\begin{array}{ccc} 0.4|\beta_{k}^{FR}||g_{k}^{T}d_{k}|+|g_{k+1}^{T}d_{k+1}|\geq \theta_{k+1}{∥g_{k+1}∥}^{2}. & & \end{array}$$ (3.6)

Recall that ${\left(a+0.4b\right)}^{2}\leq (1+0.4^{2})(a^{2}+b^{2})$ for *a*, *b* ∈ ℝ, is always true. If we apply the above condition where $a=|g_{k+1}^{T}d_{k+1}|$ and $b=|\beta_{k}^{FR}g_{k}^{T}d_{k}|$ on relation (3.6), then


$${\left(g_{k+1}^{T}d_{k+1}\right)}^{2}+{\left(\beta_{k}^{FR}\right)}^{2}{\left(g_{k}^{T}d_{k}\right)}^{2}\geq \frac{\theta_{k+1}^{2}}{1+0.4^{2}}{∥g_{k+1}∥}^{4}.$$

By denoting $c=\theta_{k+1}^{2}∕(1+0.4^{2})$, the above inequality can be expressed as


$$\begin{array}{cccc} \frac{{\left(g_{k+1}^{T}d_{k+1}\right)}^{2}}{{∥d_{k+1}∥}^{2}}+\frac{{\left(g_{k}^{T}d_{k}\right)}^{2}}{{∥d_{k}∥}^{2}} & =\frac{1}{{∥d_{k+1}∥}^{2}}[{\left(g_{k+1}^{T}d_{k+1}\right)}^{2}+\frac{{∥d_{k+1}∥}^{2}}{{∥d_{k}∥}^{2}}{\left(g_{k}^{T}d_{k}\right)}^{2}] & & \\ & \geq \frac{1}{{∥d_{k+1}∥}^{2}}[c{∥g_{k+1}∥}^{4}+{\left(g_{k}^{T}d_{k}\right)}^{2}(\frac{{∥d_{k+1}∥}^{2}}{{∥d_{k}∥}^{2}}-{\left(\beta_{k}^{FR}\right)}^{2})]. & \end{array}$$ (3.7)

In a similar process, applying (3.4) will produce


$$\frac{{∥d_{k+1}∥}^{2}}{{∥d_{k}∥}^{2}}\geq {\left(\beta_{k}^{FR}\right)}^{2}-\theta_{k+1}^{2}\frac{{∥g_{k+1}∥}^{2}}{{∥d_{k}∥}^{2}}.$$

That is,


$$\frac{{∥d_{k+1}∥}^{2}}{{∥d_{k}∥}^{2}}-{\left(\beta_{k}^{FR}\right)}^{2}\geq -\theta_{k+1}^{2}\frac{{∥g_{k+1}∥}^{2}}{{∥d_{k}∥}^{2}}.$$

This and (3.7) imply that


$$\begin{array}{cccc} \frac{{\left(g_{k+1}^{T}d_{k+1}\right)}^{2}}{{∥d_{k+1}∥}^{2}}+\frac{{\left(g_{k}^{T}d_{k}\right)}^{2}}{{∥d_{k}∥}^{2}} & \geq \frac{{∥g_{k+1}∥}^{4}}{{∥d_{k+1}∥}^{2}}[c-\theta_{k+1}^{2}\frac{{\left(g_{k}^{T}d_{k}\right)}^{2}}{{∥d_{k}∥}^{2}{∥g_{k+1}∥}^{2}}] & & \\ & =\theta_{k+1}^{2}\frac{{∥g_{k+1}∥}^{4}}{{∥d_{k+1}∥}^{2}}[\frac{1}{1+0.4^{2}}-\frac{{\left(g_{k}^{T}d_{k}\right)}^{2}}{{∥d_{k}∥}^{2}{∥g_{k+1}∥}^{2}}], & \end{array}$$ (3.8)

where $\theta_{k+1}>\frac{1.2\sigma }{1-\sigma }.$ But from Lemma 3.2, we know that


$$\underset{k\to \infty }{\lim}\frac{{\left(g_{k}^{T}d_{k}\right)}^{2}}{{∥d_{k}∥}^{2}}=0.$$

Now, if ${\mathrm{lim inf}}_{k\to \infty }∥g_{k}∥=0$ is not true, then


$$\underset{k\to \infty }{\lim}\frac{{\left(g_{k}^{T}d_{k}\right)}^{2}}{{∥d_{k}∥}^{2}{∥g_{k+1}∥}^{2}}=0.$$

Therefore, from (3.8), we get


$$\frac{{\left(g_{k+1}^{T}d_{k+1}\right)}^{2}}{{∥d_{k+1}∥}^{2}}+\frac{{\left(g_{k}^{T}d_{k}\right)}^{2}}{{∥d_{k}∥}^{2}}\geq c\frac{{∥g_{k+1}∥}^{4}}{{∥d_{k+1}∥}^{2}}.$$

It is evident that, the above description with Lemma 3.2 demonstrates that the required outcome is attained, thereby validating (3.3). ∎

**Corollary 3.4.** *Let’s assume*


$$\begin{array}{ccc} \overset{\infty }{\underset{k=1}{\sum }}\frac{1}{{∥d_{k}∥}^{2}}=\infty & & \end{array}$$ (3.9)

*is true using conclusion of Lemma 3.3. Then this implies ${\mathrm{lim inf}}_{k\to \infty }∥g_{k}∥=0$.*

*Proof.* Assume there exits a constant *𝜀* satisfying $∥g_{k}∥\geq 𝜀$ ∀*k* ≥ 1. Then, Lemma 3.3 demonstrates that (3.3) is satisfied and thus, we obtain


$$\overset{\infty }{\underset{k=1}{\sum }}\frac{1}{{∥d_{k}∥}^{2}}\leq \frac{1}{{𝜀}^{4}}\overset{\infty }{\underset{k=1}{\sum }}\frac{{∥g_{k}∥}^{4}}{{∥d_{k}∥}^{2}}<\infty$$

which contradicts (3.9) and thus, (3.2) must hold. ∎

**Lemma 3.5.** *Let $\left\{d_{k}\right\}$ follow from the proposed Algorithm 1. Suppose Assumption 3.1 is true and there is a constant* *ϱ* > 0 *satisfying $|g_{k+1}^{T}y_{k}|\geq \varrho ∥g_{k+1}∥∥s_{k}∥$, then $\left\{d_{k}\right\}$ is bounded.*

**Theorem 3.6.** *Suppose that the sequence $\left\{x_{k}\right\}$ is generated by the proposed DQSFR Algorithm where $d_{k}$ is a descent spectral direction with a seleted $\alpha_{k}$ fulfilling the SWP criteria, then the sequence $\left\{g_{k}\right\}$ generated by DQSFR Algorithm satisfies*


$$\begin{array}{ccc} \underset{k\to \infty }{\mathrm{lim inf}}∥g_{k}∥=0. & & \end{array}$$ (3.10)

*Proof.* Suppose, by contradiction, that conclusion (3.10) is not true. Then there exits $\hat{\gamma }>0$ such that $∥g_{k}∥\geq \hat{\gamma }$ for all *k* ≥ 0. From (1.7) and Assumption 3.1, we get


$$|\beta_{k}^{FR}|=\frac{{∥g_{k+1}∥}^{2}}{{∥g_{k}∥}^{2}}\leq \frac{\gamma^{2}}{{\hat{\gamma }}^{2}}:=B.$$

Now, applying (2.6) and $s_{k}=\alpha_{k}d_{k}$ together with $\alpha_{k}\geq 𝜗>0$ and $∥s_{k}∥=∥x_{k+1}-x_{k}∥\leq b$ implies


$$\begin{array}{llll} \left|\theta_{k+1}\right| & \leq |\frac{g_{k+1}^{T}s_{k}}{g_{k+1}^{T}y_{k}}+B\frac{y_{k}^{T}d_{k}}{g_{k+1}^{T}y_{k}}|\; & & \;\\ & \leq \frac{∥g_{k+1}∥∥s_{k}∥}{\varrho ∥g_{k+1}∥∥s_{k}∥}+B\frac{L∥d_{k}∥∥s_{k}∥}{\varrho ∥g_{k+1}∥∥s_{k}∥}\; & & \;\\ & =\frac{1}{\varrho }+B\frac{L∥d_{k}∥}{\varrho ∥g_{k+1}∥}\; & & \;\\ & =\frac{1}{\varrho }+B\frac{L\frac{∥s_{k}∥}{\alpha_{k}}}{\varrho ∥g_{k+1}∥}\; & & \;\\ & =\frac{1}{\varrho }+B\frac{L\frac{b}{𝜗}}{\varrho \hat{\gamma }}:=\tau .\; & & \; \end{array}$$

Combing the above inequalities with (2.1), we conclude that


$$\begin{array}{llll} ∥d_{k+1}∥ & =∥-\theta_{k+1}g_{k+1}+\beta_{k}^{FR}d_{k}∥\; & & \;\\ & \leq |\theta_{k+1}|∥g_{k+1}∥+|\beta_{k}^{FR}|∥d_{k}∥\; & & \;\\ & \leq \gamma \tau +B\frac{b}{𝜗}.\; & & \; \end{array}$$

From the relation, we have


$$\overset{\infty }{\underset{k=1}{\sum }}\frac{1}{{∥d_{k}∥}^{2}}=\infty ,$$

and this completes the proof of Corollary 3.4. ∎

**Remark 3.7** *Based on the analysis in [37,38] and the fact that $|g_{k+1}^{T}y_{k}|=∥g_{k+1}∥∥y_{k}∥|cos$ $\left\langle g_{k+1},y_{k}\rangle \right|$ and $∥y_{k}∥\leq L∥s_{k}∥,$ it is as a matter of fact, not too restrictive to select the parameter *ϱ*.*

## 4 Numerical results

In this section, we demonstrate the computational behavior of the defined DQSFR Algorithm and compare it with other notable CG methods to demonstrate its computational efficiency. All the other algorithms chosen have global convergence with their respective Wolfe line search and performance comparison is measured based on number of iterations, function evaluations, and CPU time. The obtained numerical results will support the excellent theoretical results obtained in the previous section. For the numerical evaluations, the study considered a set of 146 small and large-scale unconstrained optimization test functions (see Tables 1–5) from [35] and [39] with dimension ranging from 2 to 100,000. The algorithms used for investigating the performance are as follows:

- The proposed DQSFR Algorithm where *σ* = 0.5 and *δ* = 0.0001 in (1.3) and (1.5).
- The classical FR algorithm where direction $d_{k}=-g_{k}$ + $\beta_{k}^{FR}d_{k-1},$ and $\beta_{k}^{FR}$ follows from (1.7) with the Wolfe parameters *σ* = 0.5 and *δ* = 0.0001.
- CG_DESCENT: Hager and Zhang algorithm [40] with $d_{k}$ computed as (1.6) where $\beta_{k}$ follows from (1.14) with the Wolfe parameters *σ* = 0.5 and *δ* = 0.001.
- MSFR: Algorithm of Du and Chen[41] where $d_{k}$ follows from (1.6) and $\beta_{k}^{FR}$ as defined in (1.7) with the Wolfe parameters *σ* = 0.01 and *δ* = 0.001.
- MSTCG Algorithm of Amini and Faramarzi [42] with direction $d_{k+1}=-\theta_{k}g_{k}+\frac{g_{k}^{T}y_{k-1}}{w_{k}}d_{k-1}-\frac{g_{k}^{T}d_{k-1}}{w_{k}}y_{k-1}$, where $w_{k}=\max\{\psi ∥d_{k-1}∥∥y_{k-1}∥,d_{k-1}^{T}y_{k-1},∥g_{k-1}{∥}^{2}\}$, *ψ* = 0.2, with the Wolfe parameters *σ* = 0.1 and *δ* = 0.01 .
- TSCG Faramarzi and Amini Algorithm [31] where $d_{k}=-\theta_{k}g_{k}+\beta_{k}d_{k-1}-u_{k}z_{k-1},$ and $\beta_{k}=\frac{g_{k}^{T}z_{k-1}}{d_{k-1}^{T}z_{k-1}},$ $u_{k}=\frac{g_{k}^{T}d_{k-1}}{d_{k-1}^{T}z_{k-1}}$ with the Wolfe parameters *σ* = 0.1 and *δ* = 0.01.
- MF spectral Algorithm of Hatem et al. [43] whose direction is computed as (1.6) where $\beta_{k}$ follows from [43] with the Wolfe parameters *σ* = 0.6 and *δ* = 0.01 .

**Table 1:** Performance Results based on NOI, NOF, and CPUT.

			DQSFR			CG_DESCENT			MSTCG			MSFR			FR			MF			TSCG			
NO	Functions	DIM	Initial Guess	N0I	NOF	CPUT	N0I	NOF	CPUT	N0I	NOF	CPUT	N0I	NOF	CPUT	N0I	NOF	CPUT	N0I	NOF	CPUT	N0I	NOF	CPUT
P1	EXT PENALTY	100	(3,…,3)	7	32	0.0029	7	33	0.0010	4	24	0.0023	4	47	0.0021	7	32	0.0015	7	32	0.0020	4	24	0.0133
P2	EXT PENALTY	10000	(3,…,3)	5	24	0.0011	5	25	0.0013	4	45	0.0016	3	19	0.0022	5	24	0.0015	5	24	0.0012	4	45	0.0068
P3	EXT PENALTY	20000	(3,…,3)	5	24	0.0026	5	25	0.0022	4	46	0.0018	3	19	0.0028	5	24	0.0021	5	24	0.0017	4	46	0.0031
P4	EXT MARATOS	10	(-0.5,…,-0.5)	24	146	0.0014	14	93	0.0023	220	1009	0.0019	24	179	0.0010	129	1036	0.0010	**	**	**	25	163	0.0013
P5	EXT MARATOS	30	(-0.5,…,-0.5)	24	146	0.0005	13	67	0.0004	217	996	0.0026	24	179	0.0028	**	**	**	**	**	**	**	**	**
P6	EXT MARATOS	100	(-0.5,…,-0.5)	24	146	0.0003	1205	62493	0.0004	208	957	0.0003	24	179	0.0007	**	**	**	**	**	**	20	128	0.0005
P7	DIAGONAL 5	10	(1.1,…,1.1)	2	11	0.0364	2	12	0.0024	2	11	0.0050	2	13	0.0058	2	13	0.0037	2	13	0.0054	2	11	0.0698
P8	DIAGONAL 5	50000	(1.1,…,1.1)	2	11	0.0827	2	12	0.0819	2	11	0.0901	2	13	0.1087	2	13	0.0913	2	13	0.0931	2	11	0.1132
P9	DIAGONAL 5	100000	(1.1,…,1.1)	2	11	0.1483	2	12	0.1554	2	11	0.1586	2	13	0.2061	2	13	0.1728	2	13	0.1878	2	11	0.1860
P10	TRECANNI	2	(5,5)	11	55	0.0003	11	52	0.0004	12	75	0.0005	10	51	0.0005	11	51	0.0006	16	73	0.0003	9	47	0.0012
P11	TRECANNI	2	(50,50)	12	71	0.0004	11	68	0.0004	11	72	0.0004	11	64	0.0006	154	646	0.0003	22	108	0.0002	10	65	0.0003
P12	Q. PENALTY 1	1000	(1,…,1)	7	30	0.0199	7	30	0.0059	12	106	0.0211	7	81	0.0170	14	58	0.0096	18	74	0.0132	7	30	0.0278
P13	Q. PENALTY 1	2000	(1,…,1)	7	30	0.0070	7	30	0.0070	13	108	0.0250	**	**	**	13	54	0.0163	12	50	0.0158	**	**	**
P14	Q. PENALTY 1	5000	(1,…,1)	7	30	0.0152	**	**	**	11	75	0.0409	**	**	**	**	**	**	**	**	**	7	31	0.0250
P15	Q. PENALTY 1	7000	(1,…,1)	7	30	0.0173	**	**	**	**	**	**	**	**	**	**	**	**	7	30	0.0254	7	31	0.0265
P16	Q. PENALTY 2	50	(-3,…,-3)	8	45	0.0194	74	3361	0.3013	8	95	0.0094	**	**	**	**	**	**	**	**	**	**	**	**
P17	Q. PENALTY 2	60	(-3,…,-3)	9	147	0.0140	22	598	0.0447	**	**	**	**	**	**	**	**	**	**	**	**	13	73	0.0150
P18	QUADRATIC FUNC 1	2	(4,…,4)	2	9	0.0140	2	9	0.0035	2	9	0.0024	2	9	0.0044	2	9	0.0031	13	53	0.0028	3	13	0.0201
P19	QUADRATIC FUNC 1	6	(4,…,4)	20	144	0.0125	30	211	0.0125	17	313	0.0219	11	359	0.0217	**	**	**	14	87	0.0069	130	902	0.0869
P20	QUADRATIC FUNC 1	8	(4,…,4)	40	692	0.0366	62	995	0.0426	16	261	0.0143	36	1153	0.0624	**	**	**	22	273	0.0140	350	1817	0.1313
P21	QUADRATIC FUNC 2	50	(1,…,1)	3	13	0.0207	4	17	0.0067	2	32	0.0051	2	10	0.0030	3	13	0.0039	3	13	0.0031	2	9	0.0219
P22	QUADRATIC FUNC 2	1000	(1,…,1)	3	13	0.0026	4	17	0.0024	1	6	0.0013	1	6	0.0022	3	13	0.0026	3	13	0.0031	3	13	0.0049
P23	QUADRATIC FUNC 2	1000	(1,…,1)	3	13	0.0042	4	17	0.0071	1	6	0.0026	1	6	0.0033	3	13	0.0058	3	13	0.0059	3	13	0.0065
P24	POWER1	2	(0.5,0.5)	11	45	0.0003	15	61	0.0003	2	11	0.0004	4	25	0.0004	11	45	0.0002	12	49	0.0003	3	14	0.0004
P25	POWER1	2	(2,2)	12	49	0.0004	16	65	0.0004	2	11	0.0004	4	25	0.0005	12	49	0.0003	13	53	0.0003	3	15	0.0004
P26	ZETTLE	2	(13, -0.013)	7	44	0.0004	23	128	0.0005	28	185	0.0010	5	35	0.0003	7	44	0.0002	10	59	0.0003	5	40	0.0003
P27	ZETTLE	2	(0.5,-5)	11	55	0.0003	11	53	0.0010	26	133	0.0003	72	328	0.0003	15	73	0.0004	186	753	0.0004	11	61	0.0008
P28	DIAGONAL 2	4	(1,…,1)	12	64	0.0169	10	55	0.0055	12	65	0.0054	12	64	0.0048	12	63	0.0043	25	125	0.0091	10	54	0.0182
P29	DIAGONAL 2	6	(1,…,1)	15	82	0.0052	13	75	0.0084	13	74	0.0088	15	82	0.0074	15	82	0.0045	36	203	0.0099	17	92	0.0070
P30	DIAGONAL 2	10	(1,…,1)	21	123	0.0111	18	108	0.0087	21	120	0.0104	21	123	0.0117	21	122	0.0120	54	297	0.0228	18	108	0.0100
P31	DIAGONAL 2	12	(1,…,1)	23	136	0.0115	19	115	0.0093	24	134	0.0118	23	137	0.0162	23	135	0.0095	62	342	0.0197	23	133	0.0151
P32	TEST	3	(-7,-7,-7)	112	612	0.0437	25	150	0.0081	**	7048	0.3591	59	336	0.0272	112	616	0.0456	370	2387	0.1001	15	110	0.0238

**Table 2:** Performance Results based on NOI, NOF, and CPUT.

			DQSFR			CG_DESCENT			MSTCG			MSFR			FR			MF			TSCG			
NO	Functions	DIM	Initial Guess	N0I	NOF	CPUT	N0I	NOF	CPUT	N0I	NOF	CPUT	N0I	NOF	CPUT	N0I	NOF	CPUT	N0I	NOF	CPUT	N0I	NOF	CPUT
P33	TEST	3	(-1,-1,-1)	113	617	0.0361	24	141	0.0076	**	5997	0.3383	29	173	0.0103	167	909	0.0463	1340	7370	0.3113	18	110	0.0071
P34	SUM SQUARE1	100	(0.1,….,0.1)	12	192	0.0007	**	**	**	**	**	**	4	81	0.0005	12	192	0.0003	**	**	**	16	257	0.0004
P35	SUM SQUARE1	2000	(0.1,….,0.1)	16	399	0.0004	**	**	**	**	**	**	**	**	**	16	399	0.0004	**	**	**	**	**	**
P36	SUM SQUARE1	5000	(0.1,….,0.1)	27	809	0.0005	**	**	**	**	**	**	**	**	**	27	809	0.0004	**	**	**	**	**	**
P37	SHALLOW	1000	(11,…,11)	60	261	0.0005	16	84	0.0004	41	200	0.0004	169	738	0.0004	370	1516	0.0003	219	892	0.0005	14	70	0.0006
P38	SHALLOW	10000	(11,…,11)	62	269	0.0008	13	70	0.0009	43	208	0.0009	178	774	0.0008	395	1616	0.0009	235	956	0.0009	12	66	0.0016
P39	DQUARTC	100	(0.15,…,0.15)	4	37	0.0207	2	13	0.0047	3	25	0.0054	2	21	0.0043	3	25	0.0040	2	14	0.0038	3	25	0.0217
P40	DQUARTC	1000	(0.15,…,0.15)	4	37	0.0133	3	33	0.0111	4	45	0.0144	2	21	0.0112	4	45	0.0137	3	35	0.0118	4	45	0.0185
P41	DQUARTC	5000	(0.15,…,0.15)	4	37	0.0376	3	33	0.0393	4	45	0.0525	2	21	0.0239	4	45	0.0495	3	35	0.0416	3	26	0.0370
P42	DQUARTC	10000	(0.15,…,0.15)	4	37	0.0762	3	33	0.0734	4	45	0.1139	2	21	0.0439	4	45	0.0913	3	35	0.0835	3	27	0.0630
P43	MATYAS	2	(11,11)	1	10	0.0007	1	10	0.0009	1	10	0.0006	1	10	0.0006	1	10	0.0007	1	10	0.0008	1	10	0.0010
P44	MATYAS	2	(0.5,0.5)	1	10	0.0003	1	10	0.0004	1	10	0.0012	1	10	0.0007	1	10	0.0005	1	10	0.0003	1	10	0.0014
P45	DIAGONAL 1	2	(1,…,1)	5	21	0.0030	4	17	0.0074	9	37	0.0070	8	33	0.0027	8	33	0.0031	11	45	0.0029	5	21	0.0186
P46	DIAGONAL 1	4	(1,…,1)	13	53	0.0039	10	41	0.0029	10	41	0.0049	13	54	0.0039	13	53	0.0105	24	97	0.0051	10	41	0.0036
P47	DIAGONAL 1	10	(1,…,1)	23	94	0.0127	22	91	0.0061	24	101	0.0096	23	97	0.0079	23	94	0.0093	68	274	0.0187	28	114	0.0090
P48	DIAGONAL 1	20	(1,…,1)	34	140	0.0132	34	141	0.0110	34	141	0.0121	35	175	0.0165	36	148	0.0093	136	567	0.0425	39	160	0.0155
P49	HAGER	2	(3,…,3)	6	28	0.0002	5	22	0.0003	7	39	0.0003	7	32	0.0005	8	36	0.0004	11	47	0.0003	8	39	0.0002
P50	HAGER	4	(3,…,3)	12	52	0.0005	10	42	0.0003	11	48	0.0002	9	40	0.0009	19	80	0.0005	17	72	0.0004	10	44	0.0004
P51	HAGER	10	(3,…,3)	16	66	0.0003	13	54	0.0005	19	107	0.0003	13	56	0.0004	29	118	0.0003	27	110	0.0003	21	89	0.0003
P52	HAGER	100	(3,…,3)	26	106	0.0005	26	106	0.0005	41	215	0.0004	25	114	0.0003	26	106	0.0003	**	**	**	29	119	0.0006
P53	ZIRILLI	2	(0.30,…,0.30)	8	34	0.0003	6	26	0.0003	6	26	0.0004	51	207	0.0003	51	206	0.0004	10	43	0.0002	6	26	0.0004
P54	ZIRILLI	2	(0.2,…,0.2)	7	31	0.0003	7	31	0.0002	7	31	0.0005	142	577	0.0004	136	547	0.0002	10	44	0.0004	9	39	0.0003
P55	RAYDAN 1	10	(1,…,1)	18	101	0.0187	20	111	0.0082	18	92	0.0089	19	110	0.0105	19	109	0.0080	57	310	0.0163	17	95	0.0181
P56	RAYDAN 1	20	(1,…,1)	28	138	0.0119	27	132	0.0121	28	141	0.0112	28	139	0.0096	28	138	0.0095	116	532	0.0181	28	135	0.0086
P57	RAYDAN 1	50	(1,…,1)	47	189	0.0115	51	206	0.0107	50	203	0.0195	47	194	0.0083	47	189	0.0082	251	1005	0.0556	**	**	**
P58	RAYDAN 1	100	(1,…,1)	71	286	0.0256	66	267	0.0207	155	671	0.0655	68	301	0.0257	73	294	0.0209	567	2271	0.1749	**	**	**
P59	RAYDAN 2	10000	(0.3,…,0.3)	2	9	0.0205	2	9	0.0108	2	10	0.0108	2	9	0.0086	2	9	0.0087	2	9	0.0109	2	10	0.0288
P60	RAYDAN 2	50000	(0.3,…,0.3)	2	9	0.0266	2	9	0.0327	2	10	0.0307	2	9	0.0342	2	9	0.0363	2	9	0.0398	2	10	0.0368
P61	RAYDAN 2	100000	(0.3,…,0.3)	2	9	0.0501	2	9	0.0632	2	10	0.0641	2	9	0.0511	2	9	0.0653	2	9	0.0720	2	10	0.0693
P62	FLETCHCR	10	(0.99,…,0.99)	2	11	0.0003	2	11	0.0004	2	11	0.0004	2	11	0.0003	2	11	0.0005	2	11	0.0005	2	11	0.0003
P63	FLETCHCR	1000	(0.99,…,0.99)	2	11	0.0003	3	15	0.0004	2	11	0.0008	2	11	0.0005	2	11	0.0003	2	11	0.0004	2	11	0.0003
P64	FLETCHCR	50000	(0.99,…,0.99)	3	15	0.0032	3	15	0.0046	2	11	0.0031	3	15	0.0029	3	15	0.0043	3	15	0.0041	2	11	0.0050

**Table 3:** Performance Results based on NOI, NOF, and CPUT.

			DQSFR			CG_DESCENT			MSTCG			MSFR			FR			MF			TSCG			
NO	Functions	DIM	Initial Guess	N0I	NOF	CPUT	N0I	NOF	CPUT	N0I	NOF	CPUT	N0I	NOF	CPUT	N0I	NOF	CPUT	N0I	NOF	CPUT	N0I	NOF	CPUT
P65	DIAGONAL 3	2	(11,…,11)	9	40	0.0032	8	50	0.0060	6	40	0.0033	13	58	0.0041	70	284	0.0296	14	62	0.0057	6	35	0.0159
P66	DIAGONAL 3	4	(1,…,1)	15	61	0.0040	17	69	0.0073	17	69	0.0093	16	66	0.0052	15	61	0.0047	36	145	0.0111	16	65	0.0084
P67	DIAGONAL 3	8	(1,…,1)	25	101	0.0105	22	89	0.0064	23	93	0.0084	25	103	0.0129	25	101	0.0099	86	345	0.0303	27	109	0.0087
P68	DIAGONAL 3	10	(1,…,1)	31	126	0.0085	35	143	0.0118	35	143	0.0136	35	146	0.0139	39	158	0.0107	147	590	0.0424	44	178	0.0189
P69	EXT DENSCHNB	100	(-0.03,…,-0.03)	5	21	0.0003	5	21	0.0004	10	41	0.0004	10	43	0.0004	9	37	0.0006	9	37	0.0007	5	22	0.0004
P70	EXT DENSCHNB	5000	(-0.03,…,-0.03)	5	21	0.0006	6	25	0.0006	8	37	0.0006	9	39	0.0009	7	29	0.0005	6	25	0.0007	5	21	0.0007
P71	EXT DENSCHNB	10000	(-0.03,…,-0.03)	5	21	0.0006	6	25	0.0008	8	37	0.0009	9	39	0.0011	7	29	0.0011	6	25	0.0010	5	21	0.0015
P72	DIAGONAL 6	10000	(-1.01,…,-1.01)	2	10	0.0207	3	14	0.0189	2	10	0.0097	2	11	0.0116	2	10	0.0108	2	11	0.0118	2	10	0.0231
P73	DIAGONAL 6	5000	(-1.01,…,-1.01)	2	10	0.0054	3	14	0.0126	2	10	0.0056	2	11	0.0070	2	10	0.0076	2	11	0.0081	2	10	0.0071
P74	DIAGONAL 6	10000	(-1.03,…,-1.03)	2	10	0.0091	3	14	0.0188	2	10	0.0094	2	11	0.0097	2	10	0.0101	2	11	0.0116	2	10	0.0095
P75	DIAGONAL 6	50000	(-1.03,…,-1.03)	2	10	0.0332	3	14	0.0643	2	10	0.0390	2	11	0.0366	2	10	0.0398	2	11	0.0443	2	10	0.0432
P76	DIAGONAL 4	1000	(1,…,1)	2	9	0.0161	2	9	0.0033	2	9	0.0026	2	9	0.0032	2	9	0.0033	9	37	0.0055	3	13	0.0194
P77	DIAGONAL 4	10000	(1,…,1)	2	9	0.0050	2	9	0.0053	2	9	0.0156	2	9	0.0057	2	9	0.0071	9	37	0.0213	3	13	0.0145
P78	DIAGONAL 4	100000	(1,…,1)	2	9	0.0396	2	9	0.0444	2	9	0.0436	2	9	0.0382	2	9	0.0426	11	45	0.2429	3	13	0.0721
P79	DIAGONAL 7	10	(1,…,1)	3	13	0.0149	3	13	0.0081	3	13	0.0026	2	10	0.0022	3	13	0.0035	3	13	0.0025	3	13	0.0160
P80	DIAGONAL 7	100	(1,…,1)	3	13	0.0023	3	13	0.0019	3	13	0.0029	2	10	0.0028	3	13	0.0016	3	13	0.0017	3	13	0.0028
P81	DIAGONAL 7	100	(1,…,1)	3	13	0.0018	3	13	0.0017	3	13	0.0030	2	10	0.0023	3	13	0.0021	3	13	0.0019	3	13	0.0020
P82	DIAGONAL 8	100	(-0.5,…,-0.5)	3	14	0.0156	3	14	0.0034	3	36	0.0032	2	11	0.0024	3	14	0.0023	3	14	0.0024	3	36	0.0176
P83	DIAGONAL 8	500	(-0.5,…,-0.5)	3	14	0.0038	3	14	0.0035	3	36	0.0091	2	11	0.0034	3	14	0.0029	3	14	0.0030	3	14	0.0042
P84	DIAGONAL 9	4	(3,…,3)	82	424	0.0485	1033	4575	0.3072	**	**	**	35	168	0.0158	**	**	**	349	1397	0.1276	**	**	**
P85	DENSCHNA	1000	(6,…,6)	17	125	0.0009	11	64	0.0005	15	90	0.0005	24	122	0.0008	59	633	0.0006	51	239	0.0007	19	124	0.0010
P86	DENSCHNA	3000	(6,…,6)	17	125	0.0008	11	63	0.0006	15	90	0.0007	24	122	0.0007	57	625	0.0008	51	239	0.0007	19	113	0.0010
P87	DENSCHNA	15000	(6,…,6)	17	125	0.0019	9	55	0.0028	17	98	0.0021	26	130	0.0022	57	625	0.0023	53	247	0.0024	22	116	0.0021
P88	DENSCHNA	20000	(1,1)	17	125	0.0023	9	55	0.0028	17	98	0.0032	26	130	0.0027	57	625	0.0032	53	247	0.0027	19	104	0.0027
P89	DENSCHNA	50000	(1,1,1,1)	17	125	0.0050	9	53	0.0058	17	98	0.0058	26	130	0.0061	57	625	0.0068	55	255	0.0059	22	130	0.0057
P90	BLOCK DIAGONAL	100	(0.5,…,0.5)	16	67	0.0003	22	91	0.0005	13	187	0.0004	12	242	0.0004	18	75	0.0005	16	67	0.0004	**	**	**
P91	BLOCK DIAGONAL	1000	(0.5,…,0.5)	17	71	0.0005	24	99	0.0008	13	181	0.0005	13	261	0.0004	20	83	0.0008	17	71	0.0005	**	**	**
P92	BLOCK DIAGONAL	10000	(0.5,…,0.5)	18	75	0.0012	25	103	0.0017	14	189	0.0013	13	249	0.0017	21	87	0.0010	19	79	0.0015	**	**	**
P93	HIMMELBH	100	(0.3,…,0.3)	11	45	0.0003	5	21	0.0004	5	21	0.0004	11	46	0.0003	11	45	0.0008	21	85	0.0005	5	21	0.0003
P94	HIMMELBH	1000	(0.3,…,0.3)	12	49	0.0006	5	21	0.0005	5	21	0.0006	12	63	0.0006	12	49	0.0004	23	93	0.0006	5	21	0.0007
P95	HIMMELBH	5000	(0.3,…,0.3)	12	50	0.0012	5	21	0.0006	5	21	0.0011	**	**	**	**	**	**	**	**	**	5	21	0.0007
P96	HIMMELBH	10000	(0.3,…,0.3)	14	155	0.0010	5	21	0.0010	5	21	0.0010	**	**	**	14	155	0.0009	**	**	**	5	21	0.0011
P97	DQDRTIC	1000	(2,…,2)	54	1505	0.0006	261	1045	0.0004	222	7629	0.0003	**	**	**	**	**	**	400	8193	0.0005	68	273	0.0003

**Table 4:** Performance Results based on NOI, NOF, and CPUT.

			DQSFR			CG_DESCENT			MSTCG			MSFR			FR			MF			TSCG			
NO	Functions	DIM	Initial Guess	N0I	NOF	CPUT	N0I	NOF	CPUT	N0I	NOF	CPUT	N0I	NOF	CPUT	N0I	NOF	CPUT	N0I	NOF	CPUT	N0I	NOF	CPUT
P98	DQDRTIC	10000	(2,…,2)	45	1435	0.0008	195	781	0.0017	216	8249	0.0010	**	**	**	**	**	**	319	6561	0.0012	72	289	0.0010
P99	DQDRTIC	50000	(2,…,2)	38	1312	0.0031	232	929	0.0038	222	8974	0.0044	**	**	**	**	**	**	524	9503	0.0045	80	321	0.0038
P100	DQDRTIC	100000	(2,…,2)	52	1535	0.0060	222	889	0.0083	149	4704	0.0100	**	**	**	**	**	**	570	10795	0.0183	76	305	0.0061
P101	QUARTICM	1000	(2,…,2)	3	27	0.0005	3	30	0.0007	2	18	0.0009	3	35	0.0006	3	35	0.0007	3	35	0.0006	4	30	0.0005
P102	QUARTICM	10000	(2,…,2)	3	27	0.0015	3	30	0.0017	3	39	0.0019	3	35	0.0016	3	35	0.0016	3	35	0.0017	4	38	0.0015
P103	QUARTICM	50000	(3,…,3)	3	23	0.0058	4	49	0.0071	3	30	0.0071	3	46	0.0052	3	23	0.0057	4	42	0.0064	3	31	0.0057
P104	QUARTICM	100000	(3,…,3)	3	23	0.0116	4	39	0.0142	3	30	0.0150	3	46	0.0110	3	23	0.0110	4	42	0.0112	3	30	0.0104
P105	LINEAR PERTURBED	5000	(13,…,13)	2	9	0.0006	2	9	0.0005	2	9	0.0006	2	9	0.0010	2	9	0.0007	2	9	0.0005	2	9	0.0005
P106	LINEAR PERTURBED	10000	(13,…,13)	3	13	0.0008	3	13	0.0008	3	13	0.0008	3	13	0.0012	3	13	0.0011	2	9	0.0006	2	9	0.0012
P107	LINEAR PERTURBED	50000	(13,…,13)	3	13	0.0024	3	13	0.0022	3	13	0.0030	3	13	0.0025	3	13	0.0022	3	13	0.0039	3	13	0.0028
P108	LINEAR PERTURBED	100000	(13,…,13)	4	17	0.0041	4	17	0.0047	4	17	0.0048	3	13	0.0038	4	17	0.0043	3	13	0.0050	3	13	0.0050
P109	TRIG WHITE-HOLST	2	(1.5,1.5)	23	110	0.0006	13	74	0.0005	14	117	0.0004	24	118	0.0004	36	154	0.0004	**	**	**	20	105	0.0003
P110	TRIG WHITE-HOLST	2	(0.5,0.5)	14	64	0.0005	16	75	0.0003	74	302	0.0003	35	155	0.0003	28	116	0.0004	**	**	**	11	57	0.0004
P111	ENGVAL1	100	(2,…,2)	25	109	0.0006	25	112	0.0004	26	162	0.0005	23	**	**	40	281	0.0004	35	147	0.0004	**	**	**
P112	ENGVAL1	1000	(2,…,2)	32	153	0.0004	25	111	0.0004	**	**	**	**	**	**	291	11884	0.0003	31	130	0.0007	**	**	**
P113	ENGVAL1	7000	(2,…,2)	27	118	0.0009	152	6398	0.0010	**	**	**	**	**	**	**	**	**	**	**	**	**	**	**
P114	ENGVAL1	7500	(2,…,2)	34	489	0.0013	481	21777	0.0008	**	**	**	**	**	**	**	**	**	**	**	**	**	**	**
P115	ARWHEAD	2	(1,…,1)	26	343	0.0002	223	4265	0.0003	25	387	0.0003	**	**	**	**	**	**	**	**	**	**	**	**
P116	ARWHEAD	2	(11,…,11)	27	514	0.0004	**	**	**	7	90	0.0003	**	**	**	**	**	**	**	**	**	**	**	**
P117	DENSCHNF	1000	(3,…,3)	11	63	0.0006	10	60	0.0004	17	86	0.0004	34	167	0.0005	15	79	0.0004	16	83	0.0004	13	74	0.0005
P118	DENSCHNF	10000	(3,…,3)	11	63	0.0015	9	55	0.0013	19	94	0.0024	37	179	0.0029	16	83	0.0012	17	87	0.0018	14	80	0.0026
P119	DENSCHNF	50000	(3,…,3)	11	63	0.0059	9	55	0.0090	19	94	0.0080	37	179	0.0092	16	83	0.0063	17	87	0.0060	14	80	0.0054
P120	DENSCHNF	100000	(3,…,3)	11	63	0.0110	9	55	0.0124	19	94	0.0121	38	183	0.0200	16	83	0.0116	18	91	0.0180	14	79	0.0118
P121	DENSCHNF	200000	(3,…,3)	11	63	0.0211	9	55	0.0226	19	94	0.0272	38	183	0.0406	16	83	0.0249	18	91	0.0268	14	79	0.0250
P122	DENSCHNF	500000	(3,…,3)	11	63	0.0932	9	55	0.1042	19	94	0.1088	40	191	0.1308	16	83	0.1284	18	91	0.0968	15	87	0.0970
P123	ROTATED ELLIPSE 2	2	(1, 1)	1	5	0.0007	1	5	0.0008	1	5	0.0006	1	5	0.0005	1	5	0.0005	1	5	0.0008	1	5	0.0006
P124	SIX HUMP	2	(9,9)	14	92	0.0005	13	82	0.0004	12	78	0.0004	13	86	0.0003	19	112	0.0014	16	100	0.0004	22	181	0.0003
P125	SIX HUMP	2	(19,19)	14	108	0.0003	11	81	0.0003	13	94	0.0003	98	444	0.0004	1197	8702	0.0003	19	119	0.0003	14	142	0.0003
P126	SIX HUMP	2	(0.01,0.01)	28	240	0.0005	9	49	0.0005	8	37	0.0003	171	1784	0.0003	205	1838	0.0003	16	66	0.0003	10	52	0.0003
P127	PRICE 4	2	(-0.03, -0.03)	2	10	0.0003	2	10	0.0002	2	11	0.0003	1	7	0.0003	2	10	0.0003	2	10	0.0003	2	11	0.0004
P128	PRICE 4	2	(-0.01, -0.01)	2	9	0.0003	2	9	0.0002	2	10	0.0003	2	9	0.0003	2	9	0.0003	2	9	0.0004	2	10	0.0003
P129	ALUFFI PENTINI	2	(0.31, 0.31)	7	30	0.0002	6	26	0.0003	6	27	0.0004	46	187	0.0003	46	186	0.0003	10	43	0.0003	6	26	0.0003

**Table 5:** Numerical result of DQSFR, CG-DESCENT, MSTCG, MSFR, FR, MF, and TSCG methods.

			DQSFR			CG_DESCENT			MSTCG			MSFR			FR			MF			TSCG			
S/N	Test Functions	Dimm	Initial Point	NI	FE	CPU	NI	FE	CPU	NI	FE	CPU	NI	FE	CPU	NI	FE	CPU	NI	FE	CPU	NI	FE	CPU
P130	ALUFFI PENTINI	2	(0.29, 0.29)	8	34	0.0002	6	26	0.0003	7	30	0.0005	55	223	0.0003	55	222	0.0003	10	43	0.0006	6	26	0.0003
P131	EXT HIMMELBLAU	5000	(3,…,3)	70	1744	1.1454	60	298	0.2367	38	935	0.5716	**	**	**	**	**	**	43	413	0.3027	**	**	**
P132	EXT HIMMELBLAU	8000	(3,…,3)	102	2458	2.1964	61	322	0.3723	31	867	0.8061	**	**	**	**	**	**	44	461	0.4864	**	**	**
P133	EXT HIMMELBLAU	10000	(3,…,3)	66	1420	1.5292	61	302	0.3639	33	746	0.9136	**	**	**	**	**	**	42	386	0.4241	**	**	**
P134	EXT HIMMELBLAU	20000	(3,…,3)	61	1349	3.0161	62	328	0.8504	31	775	2.0230	**	**	**	**	**	**	42	357	0.8609	**	**	**
P135	EXT HIMMELBLAU	28000	(3,…,3)	64	1452	5.0164	62	305	1.1215	31	802	2.8930	**	**	**	**	**	**	41	400	1.5829	**	**	**
P136	EXT HIMMELBLAU	30000	(3,…,3)	57	1370	4.4315	62	305	1.2409	31	790	3.0353	**	**	**	**	**	**	42	378	1.5070	**	**	**
P137	El-Attar-Vidyasagar-Dutta	2	(11,11)	15	163	0.0003	16	131	0.0003	16	117	0.0004	43	266	0.0007	**	**	**	**	**	**	20	145	0.0003
P138	El-Attar-Vidyasagar-Dutta	2	(13,13)	17	121	0.0003	12	94	0.0003	16	116	0.0004	**	**	**	**	**	**	31	168	0.0003	20	147	0.0004
P139	EXT HIEBERT	2	(11,11)	45	314	0.0005	51	322	0.0004	77	580	0.0002	186	1160	0.0003	263	1175	0.0003	**	**	**	28	191	0.0002
P140	EXT HIEBERT	2	(13,13)	47	268	0.0004	36	237	0.0003	612	3291	0.0003	113	785	0.0003	46	283	0.0004	**	**	**	28	196	0.0004
P141	EXT TRIDIAGONAL 1	20000	(5,…,5)	203	927	0.0026	9	57	0.0034	437	4203	0.0031	60	305	0.0028	203	927	0.0026	273	2296	0.0030	25	117	0.0036
P142	EXT TRIDIAGONAL 1	40000	(5,…,5)	231	1052	0.0047	9	57	0.0067	539	5189	0.0063	77	385	0.0059	231	1052	0.0046	341	2874	0.0062	17	91	0.0054
P143	EXT TRIDIAGONAL 1	50000	(5,…,5)	259	1178	0.0051	9	57	0.0082	581	5595	0.0075	77	385	0.0075	259	1178	0.0059	367	3095	0.0091	25	118	0.0071
P144	EXT TRIDIAGONAL 1	100000	(5,…,5)	291	1321	0.0114	11	83	0.0163	725	6987	0.0166	78	389	0.0167	291	1321	0.0139	457	3860	0.0176	22	111	0.0167
P145	THREE HUMP	2	(3,3)	16	130	0.0003	28	138	0.0004	19	451	0.0004	**	**	**	**	**	**	24	101	0.0003	**	**	**
P146	THREE HUMP	2	(13,13)	16	130	0.0012	28	138	0.0009	19	451	0.0007	**	**	**	**	**	**	24	101	0.0009	**	**	**

All codes for the computational procedures are written in MATLAB R2023a programming software and implemented on an Intel(R) Core i7 PC with 8GB RAM, 2.90 GHz CP and XP operation system. The iteration process would be terminated if $∥g({\text{x}}_{k})∥<10^{-6},$ or any of the following holds:

1. Iterations exceed 2000 without obtaining any ${\text{x}}_{k}$ satisfying $∥g({\text{x}}_{k})∥<10^{-6}$.
2. If the direction is not descent.
3. Failure occurs due to insufficient memory during code execution.

If any of the above occur, a failure will be declared at the point and would be denoted by (**).

### 4.1 Computational efficiency of DQSFR compared with existing methods

The computational efficiency of the CG algorithms makes it particularly well-suited for solving large-scale unconstrained optimization problems where explicit storage of the Hessian matrix is prohibitive or where matrix-vector products can be computed efficiently. This is usually associated to low memory requirement, iterative nature, or convergence behaviour of the algorithm. In this study, the computational efficiency of the proposed DQSFR method is evaluated based on three metrics including number of iterations (NOI), function evaluations (NOF), and computational time (CPUT) required to achieve a tolerance level of 10^–6^ or other termination criteria. All computations are carried out under strong Wolfe strategies and the results are compared with TSCG, MF, FR, MSFR, MSTCG, and CG-DESCENT using their default parameter values for the Wolfe line search. The comprehensive numerical results is demonstrated in Tables 1-5.

Based on our findings as demonstrated in Tables 1-5, we noted that the percentage of successfully solved problems by any CG algorithm depends on several factors including the type of problem being solved, the choice of initial guess, the implementation of the CG algorithm, the accuracy requirements, the selection of line search parameters, and the convergence criteria used.

### 4.2 Accuracy

The accuracy of the proposed DQSFR algorithm is evaluated by juxtaposing its computed solutions with those generated by reference CG algorithms possessing similar attributes. Accuracy is gauged through the success and failure rates of the computed solutions, illustrating a notable correlation with the reference solutions, as delineated in Table 6.

**Table 6:** Assessing the overall accuracy of the solutions obtained by the algorithms.

	DQSFR	CG_DESCENT	MSTCG	MSFR	FR	MF	TSCG
Success	100 %	95.9 %	93.8 %	81.5 %	80.8 %	85.6 %	82.9 %
Failure	0%	4.1 %	6.2 %	18.5 %	19.2 %	14.4 %	17.1 %

Table 6 describes the % of successfully solved problems recorded by each algorithm. In the case of well-conditioned benchmark functions, it is noted that the CG algorithms frequently attains high success rates, typically reaching solution points efficiently within a moderate number of iterations. However, for ill-conditioned problems, convergence may be slower, leading to a decrease in success rates. Based on the results, it is obvious to notice that the proposed method achieved 100% success rate which implies that its algorithm has been able to solve all problems considered and thus, demonstrate its efficiency and numerical stability for large scale unconstrained optimization problems. The success of DQSFR might be associated to the conjugacy relaxation of double truncated property associated with earlier spectral methods. The other algorithms also considered achieved higher % of success rate with the classical FR method recording the highest percentage (19.2%) of failure rate. This can be attributed to the search direction failing to navigate effectively through the landscape and thus, result in stagnation or slow convergence towards a sub-optimal solution.

### 4.3 Convergence behavior

The convergence behavior of the DQSFR method is examined in this section, considering factors such as the nature of the objective function, the dimensionality of the problem and the algorithmic parameters. A well-established tool introduced by Dolan and Moré [44] is utilized to analyze the efficiency of CG algorithms through performance profiles. These profiles evaluate algorithms based on metrics like the number of iterations (NOI), function evaluations (NOF), or computational time (CPUT) required to achieve a specified level of accuracy.

**Fig 1:**
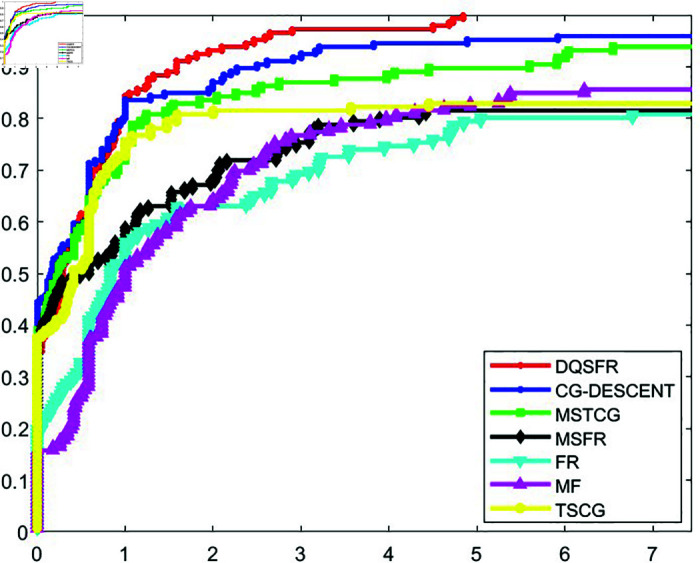
NOI performance profile.

**Fig 2:**
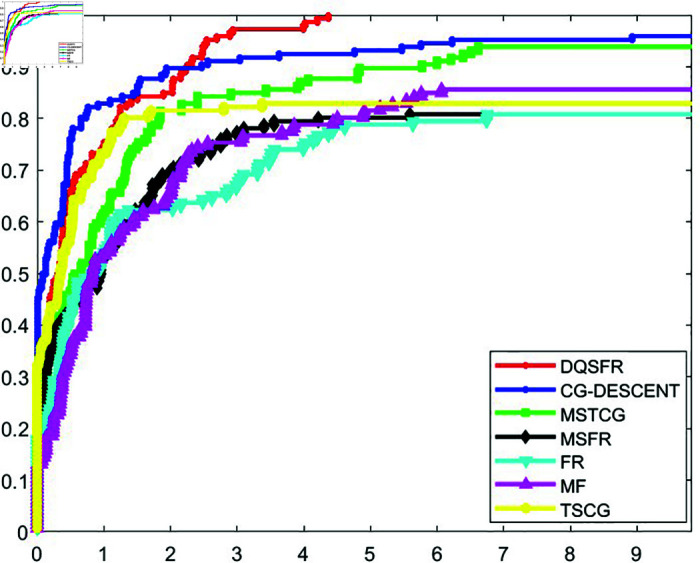
NOF performance profile.

**Fig 3:**
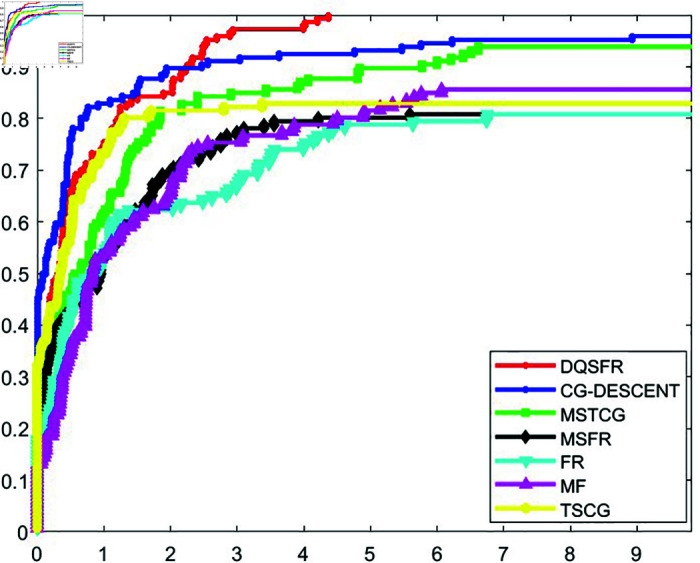
CPUT performance profile.

The performance profile defines a performance ratio for each problem *p* ∈ *P* and solver *s* ∈ *S* across a set of $n_{p}$ test problems and $n_{s}$ solvers:


rp,s=fp,s min⁡ {fp,s:s∈S and p∈P},

where the cumulative distribution function (CDF) of these ratios is plotted to compare the relative performance of algorithms. The *x*-axis represents the performance ratio, while the *y*-axis shows the fraction of problems solved at or below a given ratio. The performance profile are displayed as follow:

Figs 1, 2, and 3 display the NOI, NOF, and CPUT performance profiles, respectively, comparing the DQSFR method with CG-DESCENT, MSTCG, MSFR, FR, MF, and TSCG. The results indicate that the DQSFR method outperforms most of the algorithms across the test problems. Specifically, DQSFR achieves the highest success rate, solving 100% of the problems, which reflects its robustness and scalability. However, it is important to clarify that this success rate pertains to the specific test set and does not imply universal convergence.

Further analysis shows that the curves of CG-DESCENT, MSTCG, and TSCG methods compete closely with DQSFR at certain points. Meanwhile, the FR, MSFR, and MF methods exhibit lower performance, with curves consistently below the leading methods, indicating their limited effectiveness for the problems considered.

The observed behavior of DQSFR on high-dimensional problems is influenced by the problem characteristics. Instances of non-convergence for competing methods often arise from numerical difficulties, which are less impactful for DQSFR due to its efficient handling of such cases.

Based on the results in Tables 1–6 and the performance profiles in Figs 1–3, the DQSFR method demonstrates a compelling combination of numerical stability, efficiency, and scalability, making it a robust choice for large-scale unconstrained optimization.

### 4.4 Application to portfolio selection

Portfolio selection refers to a collection of stocks or assets owned by investors. An investor would always want to find the best way of allocating their portfolio so as to maximize profit or minimize risk [6,45]. Alternatively, one may look at maximizing the returns while incurring some risk [46,47]. In recent years, the application of conjugate gradient methods to portfolio selection problems has gained significant attention. Several studies have explored this approach, showcasing its potential in solving optimization problems within the context of portfolio management. Although classical CG methods are widely used due to their simplicity, low memory requirements, and strong convergence properties, they face significant limitations in the context of portfolio optimization. These limitations include sensitivity to ill-conditioning, slow convergence in highly irregular risk-return landscapes, and difficulties in handling non-smooth or dynamic constraints commonly encountered in real-world portfolios. As a result, the effectiveness of classical CG methods in practical portfolio optimization is often hindered. Therefore, there is a need for new CG methods that can address these challenges, providing improved computational efficiency and robustness for solving portfolio optimization problems [5,48–50].

In this study, we apply our presented method, a conjugate gradient method, together with the other competing methods, to solve a problem of risk management of a portfolio of stocks. Given a stork $r_{i},\;\;1\leq i\leq q$, its return at time *t*, denoted by $r_{it}$ is obtained as


$$\begin{array}{lll} r_{it}=\frac{P_{t}-P_{t-1}}{P_{t-1}}, & \; & \end{array}$$

where $P_{t}$ and $P_{t-1}$ denote the closing price at time *t* and *t*–1, respectively. Using the returns $r_{it}$, the mean returns, expected returns and covariance between stocks can be calculated. In solving a portfolio problem, one requires to solve the risk-averse portfolio problem


$$\begin{array}{llll} \min & \;\;\;\sigma_{p}^{2}=Y^{T}VY\; & & \;\\ \text{s.t. } & \;\;\;\overset{q}{\underset{j=1}{\sum }}w_{j}=1,\; & & \; \end{array}$$

where $Y=\{w_{1},w_{2},\ldots ,w_{q}\}$ represents the portfolio investment weighted proportions of the stocks and *V* is a *q* × *q* covariance matrix of the stocks. By setting


$$\begin{array}{lll} w_{q}=1-\overset{q-1}{\underset{j=1}{\sum }}w_{j}, & \; & \end{array}$$

the above constrained optimization problem can be converted into an unconstrained optimization problem


$$\begin{array}{ccc} \underset{Y\in {\mathbb{R}}^{q-1}}{\min}\;\;\;Y^{T}VY, & & \end{array}$$ (4.1)

which we can now easily solve using the new method and the other earlier conjugate gradient methods used for comparison.

For numerical experiments, we use a portfolio of ten (10) stocks (i.e. *q* = 10). We use weekly closing prices of stocks over a period spanning from 10 July 2023 to 10 July 2024 and these are obtained from the database https://www.investing.com/. The stocks chosen are presented in Table 7. Tables 8 and 9 show the mean of returns of the stocks and covariance between the stocks, respectively. We solve the unconstrained problem (4.1) with the covariance matrix *V* as in Table 9. We solve the problem starting with initial points $w^{0}=(0.1,0.1,\ldots ,0.1),\;\;(0.2,0.2,\ldots ,0.2),\;\;(0.3,0.3,\ldots ,0.3)$ and (0.02, 0.02, …, 0.02), where $w^{0}\in {\mathbb{R}}^{9}.$

**Table 7:** Table of companies.

LLY - Eli Lilly and Company	HBAN - Huntington Bancshares Inc.
NVDA - NVIDIA Corporation	LCID - Lucid Group, Inc.
UNH - UnitedHealth Group Inc.	AAL - American Airlines Group Inc.
JNJ - Johnson and Johnson	AMD - Advanced Micro Devices Inc.
PLTR - Palantir Technologies Inc	PFE - Pfizer Inc.

**Table 8:** Table of mean of returns.

Stock	Mean	Stock	Mean
LLY	0.003212531604056	AMD	0.002269449081475
UNH	0.000401575323838	HBAN	0.000995808604515
JNJ	-0.000128786120801	LCID	-0.002867749970768
NVDA	0.004936603934554	PFE	-0.000711600539937
PLTR	0.002841707827834	AAL	-0.001857819967647

**Table 9:** Table of covariance between stocks ($\times 10^{-3}$).

Stock	LLY	UNH	JNJ	NVDA	PLTR	AMD	HBAN	LCID	PFE	AAL
LLY	0.34681	0.009186	0.01137	0.12583	0.05833	0.03541	-0.02956	0.04974	-0.00803	-0.03040
UNH	0.00919	0.19095	0.01943	-0.05360	0.02564	-0.02332	0.00604	-0.00767	0.02798	0.04270
JNJ	0.01137	0.01943	0.10305	-0.06383	-0.00776	-0.04287	0.03533	0.02563	0.05756	0.00844
NVDA	0.12583	-0.05360	-0.06383	0.81967	0.33574	0.45520	0.01646	-0.04545	-0.04988	0.10493
PLTR	0.05833	0.02564	-0.00776	0.33574	1.69831	0.35929	0.19151	0.70339	0.07636	0.29036
AMD	0.03541	-0.02332	-0.04287	0.45520	0.35929	0.82880	0.09891	0.04030	-0.02549	0.17427
HBAN	-0.02956	0.00604	0.03533	0.01646	0.19151	0.09891	0.29143	0.28515	0.04257	0.14338
LCID	0.04974	-0.00767	0.02563	-0.04545	0.70339	0.04030	0.28515	2.02105	0.08981	0.26933
PFE	-0.00803	0.02798	0.05756	-0.04988	0.07636	-0.02549	0.04257	0.08981	0.23727	0.05579
AAL	-0.03040	0.04270	0.00844	0.10493	0.29036	0.17427	0.14338	0.26933	0.05579	0.57856

The resulting stock allocations for each of these initial points are summarized in Table 10, leading to a portfolio risk of $\sigma_{p}^{2}=0.000477$ and an expected portfolio return of 0.000747. Notably, the allocation includes a negative weight for PLTR (-1.9%), indicating a short-selling position, where the investor sells a stock they do not own, often borrowed from a broker. This allocation strategy demonstrates the flexibility of the proposed method to handle diverse investment scenarios. The results also highlight that the proposed method consistently achieves feasible and efficient solutions, demonstrating its superiority over competing methods by attaining a balance between lower risk and higher return. However, its sensitivity to initial points, which is common among iterative optimization methods, suggests the potential for further improvement, such as the inclusion of regularization techniques to enhance robustness. These findings underline the practical utility of the proposed method in portfolio optimization scenarios.

**Table 10:** Table of Stock allocation.

Stock	Allocation (%)	Stock	Allocation (%)
LLY	10.2	AMD	3.2
UNH	21.4	HBAN	9.3
JNJ	38.7	LCID	0.5
NVDA	7.8	PFE	8.9
PLTR	-1.9	AAL	1.9

## 5 Conclusion

In this work, we apply the second strong Wolfe line search criteria to show the sufficient descent of a spectral FR (DQSFR) method for large-scale unconstrained optimization problems. The method was able to relax the double truncated property associated with earlier spectral methods. This was achieved by utilizing nice convergence property of the quasi-Newton method and the extended conjugacy condition. Similarly, convergence of the new scheme is achieved without enforcing any bounded condition. Thus, to evaluate the efficiency of DQSFR method with TSCG, MF, MSTCH, CG-DESCENT, FR, and MSFR conjugate gradient algorithms, the study performed an extensive numerical experiments with the Wolfe conditions. Findings from the numerical computation shows that the proposed DQSFR method offers a compelling combination of adaptability, numerical stability, efficiency, and scalability, making it a preferred choice for solving large-scale unconstrained optimization problems in various scientific and engineering applications. In the realm of portfolio selection, the proposed method was expanded to tackle the challenge of stock allocation, maximizing returns while minimizing risks. The method utilizes the extended conjugacy condition and the favorable convergence characteristics of the quasi-Newton method to ease the double truncated property seen in previous spectral methods
